# Diarrheal Illnesses and Their Association With Hygiene Practices and Food and Water Sources Among Domestic Pilgrims During the 2022 Hajj Season

**DOI:** 10.7759/cureus.99335

**Published:** 2025-12-15

**Authors:** Mohammad O Alrukban, Abdulrahman Y Alhoumaily, Khalid M Alhamdi, Mansour A Aldhalaan, Abdullah N Abdulrazaq, Salem M Abokhanjar, Hanan A Habib

**Affiliations:** 1 Department of Family Medicine, King Saud University, Riyadh, SAU; 2 College of Medicine, King Saud University, Riyadh, SAU; 3 Department of Pathology and Laboratory Medicine, King Saud University, Riyadh, SAU

**Keywords:** diarrhea, gastrointestinal infection, hajj, mass gatherings, traveler's diarrhea

## Abstract

Objectives

This study aimed to estimate the prevalence of diarrhea and its associated symptoms among domestic pilgrims in 2022, as well as to correlate hand hygiene measures and food and water sources with the occurrence of diarrhea and its associated symptoms during the 2022 Hajj season.

Methods

An analytical cross-sectional study was carried out in Makkah, Saudi Arabia, on domestic pilgrims during the 2022 Hajj season. The data collection form was built based on a literature review and adopting a previously validated questionnaire. An online survey was used after using multi-stage cluster sampling. Data was analyzed using IBM SPSS Statistics for Windows, V. 26.0 (IBM Corp., Armonk, NY, USA). Descriptive statistics were used to describe the categorical and quantitative variables.

Results

The study has 1193 participants, aged 18-65 years. The prevalence of diarrhea was 386 (32.4%), with fever being the most reported associated symptom (192, 49.7%). When compared to other food and water sources, the majority of responders who consumed campaign-provided food and bottled water had a lower prevalence of diarrhea. Diarrhea was less common among pilgrims who washed their hands more often. Individuals who cleaned ≥5 times had the lowest frequency (117, 23.1%). Consumption of expired food and the occurrence of diarrhea were statistically significant. Statistically significant associations (p<0.0001; 95% CI) were found between diarrhea prevalence and hand hygiene frequency, food sources, water sources, and sharing behaviors.

Conclusion

Our study found a diarrhea prevalence of 32.4% among domestic pilgrims during the 2022 Hajj season, which is higher than rates reported in previous studies (1-23%). Domestic pilgrims reported varying practices related to food consumption, water sources, and hand hygiene. Discrepancies were noted between the different types of campaigns. Further qualitative research is required to provide insight into these differences.

## Introduction

Mass gatherings, such as Hajj, are defined by the World Health Organization (WHO) as "events attended by a sufficient number of people to strain the planning and response resources of a community, state or nation" [1]. Every year, millions of people come to The Holy Mosque in Makkah, Saudi Arabia, for Hajj, making Hajj the largest annual mass gathering. People from different ethnicities, nationalities, age groups, and educational backgrounds visit Makkah to perform Hajj, which can only be performed during a certain period of seven days every lunar year on the land of Makkah; such a large assembly creates an ideal environment for infectious diseases [2,3].

Historical outbreaks during Hajj, including cholera and meningococcal disease, highlight the ongoing infection risks associated with mass gatherings [3,4].

Due to the dense population and tight quarters, gastrointestinal (GI) diseases can occur often during the Hajj season [5]. Common reported GI symptoms were diarrhea, abdominal pain, nausea, and vomiting [5,6]. Diarrhea is one of the most reported symptoms during the Hajj season. It is defined by WHO as "the passage of three or more loose or liquid stools per day, or more frequent passage than is normal for the individual" [7].

GI illnesses were ranked as the third most frequent reason for pilgrims to seek medical attention in Mina during the 2008 Hajj season, according to a study that examined the patterns of disorders among these individuals. According to the study, this might be related to inadequate food sanitization standards [8]. Consuming street food or communal meals may increase the risk of foodborne illnesses, resulting in nausea, vomiting, diarrhea, and stomach cramps [5].

It is thought that maintaining good hand hygiene is essential for preventing diarrhea [7,9]. Given its ability to reduce self-reported respiratory infections, hand hygiene is thought to be the most popular infection prevention strategy among pilgrims [10,11]. The prevalence of diarrhea can be considerably reduced by drinking safe water and maintaining good hygiene. Moreover, ingestion of contaminated food may contribute to the development of a wide range of diseases, including diarrhea [12-14].

The COVID-19 pandemic led to an increased awareness regarding sanitization and hand hygiene among the public [15]. However, to our knowledge, no research on the impact of these hygiene practices on the prevalence of diarrhea during the Hajj has been carried out since the COVID-19 pandemic. Therefore, this study aims to estimate the prevalence of diarrhea and its associated symptoms among domestic pilgrims and correlate hand hygiene measures and food and water sources with the existence of diarrhea. The choice of selecting only domestic pilgrims is due to the nature of Hajj logistics, as domestic pilgrims arriving from different parts of Saudi Arabia are allocated to different camps organized in three clusters based on cost. Camps and the number of individuals are registered with the National Committee of Hajj and Umrah. This allows more accurate sample size calculation and sampling technique.

## Materials and methods

This analytical cross-sectional study was conducted during the Hajj season in Makkah, Saudi Arabia, from July 9 to 14, 2022. In that year, the Hajj was controlled by COVID-19 restriction measures in terms of the number of pilgrims and their age. This study focused on domestic pilgrims as they represent a distinct population with unique characteristics and accessibility for longitudinal follow-up compared to international pilgrims who depart immediately after Hajj. Domestic pilgrims were approximately 119,000 individuals, and their ages ranged between 18 and 65 years.

Sampling strategy

We employed a three-stage cluster sampling design to ensure representative sampling across different accommodation types while maintaining feasibility given the logistical constraints of the Hajj mass gathering. The sampling procedure was structured as follows:

Stage 1: Cluster Stratification

The target population of domestic pilgrims was stratified into three distinct clusters based on accommodation cost categories as classified by the National Committee of Hajj and Umrah: (1) standard camps (lowest cost tier), (2) upgraded camps (mid-tier), and (3) tower class accommodations (premium tier). This stratification was chosen because accommodation type serves as a proxy for socioeconomic status and is associated with differential access to hygiene facilities and food services.

Stage 2: Campaign Selection

From the official registry maintained by the National Committee of Hajj and Umrah, we obtained comprehensive lists of all registered campaigns within each cluster stratum. Using computer-generated random numbers (Microsoft Excel RAND function (Microsoft Corp., Redmond, WA, USA)), we randomly selected four campaigns from each of the three clusters, yielding 12 campaigns total (four standard + four upgraded + four tower campaigns). Randomization was maintained through a lottery method where each campaign within a stratum had an equal probability of selection. The random seed was set at the beginning to ensure reproducibility.

Stage 3: Within-Campaign Participant Selection

Campaign managers were provided with the study protocol and asked to distribute online questionnaires to pilgrims in their respective campaigns. To minimize selection bias at this stage, questionnaires were distributed based on randomly generated participant identification numbers provided by the campaign registration system. Specifically, campaign managers were instructed to send questionnaires to approximately 100 pilgrims per campaign, targeting every nth pilgrim on their alphabetically sorted registration list (where n=total registered pilgrims/100). This systematic random sampling approach within each selected campaign ensured that participant selection remained independent of pilgrim characteristics. Questionnaire distribution was automated through an online platform that recorded no more than the required number of responses per campaign, preventing oversampling from any single site.

The data collection form was built based on a literature review and adopting a previously validated questionnaire after obtaining permission from the authors [5]. The questionnaire was presented to the participants in both Arabic and English language based on their preferences. It was divided into five sections. The first section was concerned with demographics which included age, gender, and nationality. The next section in the survey covered diarrhea and its associated symptoms, which were fever, vomiting, nausea, abdominal pain, and bloating. The remaining section concerned food and water resources and hand hygiene measures.

Food resources included street vendors, restaurants, cooking one's own food, and food provided by each campaign. Water resources were tap, bottled, and street water points. Hand hygiene was assessed based on daily frequency (0, less than 3, 3-5, and more than 5). General hygiene practices in relation to food and water were the sharing of plates and utensils, as well as water sharing. Consumption of expired food was also assessed.

Data was analyzed using IBM SPSS Statistics for Windows, V. 26.0 (IBM Corp., Armonk, NY, USA). Descriptive statistics (frequencies, percentages, mean, and standard deviation) were used to describe the categorical and quantitative variables. Moreover, Pearson's chi-squared test was used to test the association between categorical study and outcome variables. A p-value of ≤0.05 and 95% confidence intervals (CIs) were used to report the statistical significance and precision of the results. Additionally, odds ratios (OR) with 95% CI were calculated for all categorical variables to quantify effect sizes. To identify independent predictors of diarrhea, multivariate logistic regression analysis was performed using backward stepwise elimination. Variables with a p-value of <0.10 in univariate analysis were entered into the initial model, and non-significant variables (p≥0.05) were sequentially removed. Adjusted odds ratios (aOR) with 95% CI were reported for variables retained in the final model. Model fit was assessed using the Hosmer-Lemeshow goodness-of-fit test, and Nagelkerke R² was calculated to estimate the proportion of variance explained by the model.

The study was approved by the King Saud University Institutional Review Board (approval number: E-22-6991). Participants weren't coerced to participate; a consent form was provided at the beginning of the survey.

## Results

Among the 1218 pilgrims who were invited to participate in the study, 1193 accepted to participate. Their age group ranged between 18 and 63 years, with a median age of 34 years. Most of the participants were between 28 and 47 years of age (886, 74.3%). The majority of the participants were male, 749 (62.8%), and non-Saudi, 923 (77.4%).

Almost one-third of pilgrims sought medical assistance, and some of them consulted more than one type of medical service (Table 1). Campaign doctors were the pilgrims' first aid provider in most of the cases (11.8%). Other forms of medical assistance were hospitals, ambulances, primary healthcare centers, and pharmacists.

**Table 1:** Demographic characteristics and health status of the participating pilgrims

Variable	Category	N (%)
Age (in years)	18-27	223 (18.7)
	28-37	526 (44.1)
	38-47	360 (30.2)
	48-57	72 (6)
	58-65	12 (1)
Gender	Male	749 (62.8)
	Female	444 (37.2)
Nationality	Saudi	270 (22.6)
	Non-Saudi	923 (77.4)
Need for medical assistance	Yes	407 (34.12)
	No	786 (65.88)
Total		1193 (100)

Nearly 386 (32.4%) of pilgrims reported suffering from diarrhea during the Hajj season (Table 2). The most common related symptom among those who reported diarrhea was fever (192, 49.7%) which was followed by abdominal pain (178, 46.1%). Other symptoms which were also reported included nausea (163, 42.2%), vomiting (155, 40.2%), and bloating (153, 39.6%). Moreover, a statistically significant association was found between those who didn't have diarrhea and the presence of other associated symptoms (p<0.0001).

**Table 2:** Association between diarrhea and associated symptoms among pilgrims

Associated symptoms	Diarrhea (yes), N (%)=386 (32.4)	Diarrhea (no), N (%)=807 (67.6)	Total, N (%)=1193 (100)	Pearson's chi-squared value	P-value
Fever	192 (49.7)	172 (21.3)	364 (30.5)	99.522	<0.0001
Vomiting	155 (40.2)	144 (17.8)	299 (25)	69.207	<0.0001
Nausea	163 (42.2)	152 (18.8)	315 (26.4)	73.529	<0.0001
Abdominal pain	178 (46.1)	154 (19.1)	332 (27.8)	94.991	<0.0001
Bloating	153 (39.6)	188 (23.3)	341 (28.6)	34.156	<0.0001

All associated symptoms showed significantly elevated odds of occurring among pilgrims with diarrhea compared to those without. Fever demonstrated the strongest association (OR=3.63; 95% CI: 2.76-4.78; p<0.0001), followed by abdominal pain (OR=3.54; 95% CI: 2.69-4.66; p<0.0001), nausea (OR=3.16; 95% CI: 2.40-4.16; p<0.0001), vomiting (OR=3.08; 95% CI: 2.32-4.08; p<0.0001), and bloating (OR=2.17; 95% CI: 1.65-2.85; p<0.0001). These findings indicate that GI symptoms cluster together, with fever being particularly predictive of diarrheal illness during Hajj.

Regarding food resources, the majority of pilgrims relied on their campaign's food as their main food resource (690, 57.8%), followed by street vendors (324, 27.2%) and restaurants (263, 22%), and 164 (13.7%) cooked their own food. Notably, it was observed that pilgrims who relied on their campaign's food had the lowest prevalence of diarrhea (127, 18.4%) among other resources. Street vendors' food was associated with the highest prevalence of diarrhea (170, 52.5%) (Table 3).

**Table 3:** Distribution of food and water resources in relation to the prevalence of diarrhea

Food and water resources	Diarrhea (yes), N (%)=386 (32.4)	Diarrhea (no), N (%)=807 (67.6)	Total, N (%)=1193 (100)
Street vendor's food	170 (52.5)	154 (47.5)	324 (27.2)
Restaurant's food	120 (45.6)	143 (54.4)	263 (22)
Cooked own food	72 (43.9)	92 (56.1)	164 (13.7)
Food provided by the campaign	127 (18.4)	563 (81.6)	690 (57.8)
Tap water	103 (57.2)	77 (42.8)	180 (15.1)
Street water points	104 (32.5)	216 (67.5)	320 (26.8)
Bottled water	240 (26.3)	674 (73.7)	914 (76.6)

Bottled water was the major pilgrims' water resource, 914 (76.6%), and its consumers reported the least prevalence of diarrhea, 240 (26.3%). On the other hand, the highest rate of diarrhea was reported by pilgrims who consumed tap water (103, 57.2%) (Table 3).

Regarding food resources, street vendor's food showed the highest odds of diarrhea occurrence (OR=2.20; 95% CI: 1.62-2.99; p<0.0001), followed by restaurant food (OR=1.68; 95% CI: 1.24-2.27; p<0.001). In contrast, food provided by the campaign demonstrated a strong protective effect (OR=0.18; 95% CI: 0.13-0.24; p<0.0001), with pilgrims consuming campaign food having an 82% reduced odds of diarrhea. Cooking own food also showed lower risk (OR=0.70; 95% CI: 0.51-0.98; p=0.036). For water resources, tap water consumption was associated with the highest diarrhea risk (OR=4.39; 95% CI: 3.13-6.16; p<0.0001), followed by street water points (OR=1.36; 95% CI: 1.00-1.85; p=0.048), while bottled water appeared protective (OR=0.60; 95% CI: 0.47-0.76; p<0.0001). These findings highlight that reliance on unregulated food and water sources substantially increases diarrhea risk, while campaign-provided resources offer significant protection.

Most of the pilgrims reported cleaning their hands at least three times per day (82.72%). A statistically significant difference was found between hand cleaning frequency and diarrhea, where those who didn't clean their hands had the highest percentage of diarrhea (42, 60.9%), whereas those who cleaned their hands more than five times per day had the lowest percentage of diarrhea (117, 23.1%) (p<0.0001) (Table 4).

**Table 4:** Association between different hygiene measures and practices with diarrhea

Hygiene measures and practices	Diarrhea (yes), N (%)=386 (32.4)	Diarrhea (no), N (%)=807 (67.6)	Total, N (%)=1193 (100)	Pearson's chi-squared value	P-value
Frequency of hand cleaning per day					
0	42 (60.9)	27 (39.1)	69 (5.8)	58.764	<0.0001
<3	64 (46.7)	73 (53.3)	137 (11.4)		
3-5	163 (33.9)	318 (66.1)	481 (40.3)		
>5	117 (23.1)	389 (76.9)	506 (42.4)		
Plate and utensil sharing					
Yes	155 (40.2)	169 (20.9)	324 (27.2)	48.726	<0.0001
No	231 (59.8)	638 (79.1)	869 (72.8)		
Water sharing					
Yes	203 (52.6)	213 (26.4)	416 (34.9)	78.900	<0.0001
No	183 (47.4)	594 (73.6)	777 (65.1)		
Eating expired food					
Yes	71 (50.35)	70 (49.65)	141 (11.8)	67.140	<0.0001
No	220 (25.49)	643 (74.51)	863 (72.3)		
Maybe	95 (50.26)	94 (49.74)	189 (15.8)		

More than half of the participants had a tendency not to share plates and utensils or water with others. Sharing plates and utensils had a statistically significant relation with diarrhea (p<0.0001) (Table 4).

**Figure 1:**
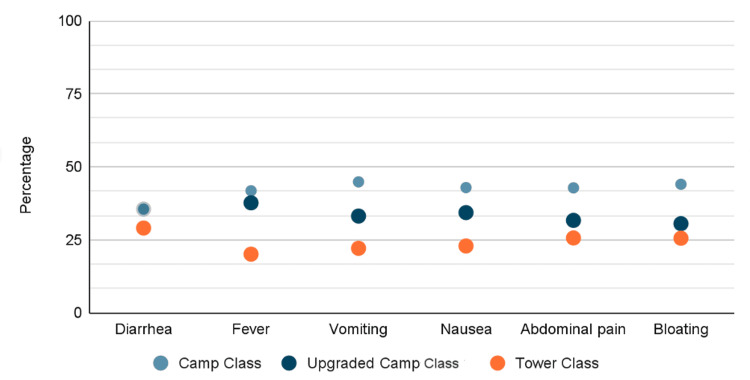
Distribution of diarrhea and its associated symptoms among different campaign classes

**Figure 2:**
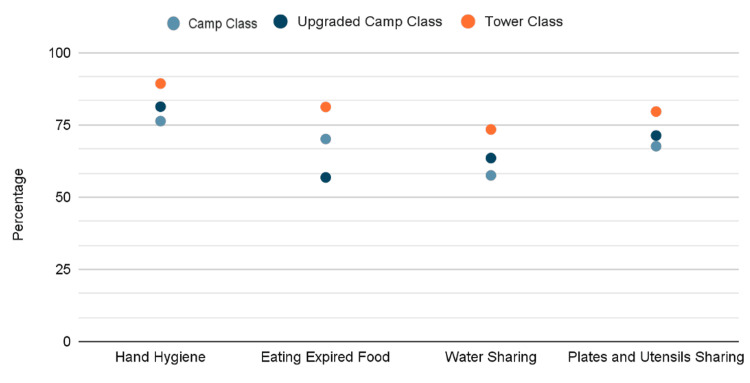
Hygiene-related practices among different campaign classes

Notably, 141 (11.8%) of participants reported eating expired food. Of those, 71 (50.4%) reported diarrhea; on the other hand, pilgrims who didn't eat expired food had the lowest percentage of diarrhea, 220 (25.5%), with a statistically significant difference (p<0.0001). The study examined the correlations between the prevalence of diarrhea and its associated symptoms in different campaign classes. The tower class campaign demonstrated the lowest prevalence of diarrhea and associated symptoms compared to other courses. Aside from diarrhea, statistically significant differences were observed in the presence of associated symptoms across various campaign classes (p<0.0001) (Figure 1). Furthermore, there was a clear association between the campaign class and adherence to healthy hygiene practices (Figure 2). Nearly 90% of pilgrims in tower campaigns reported cleaning their hands at least three times, in contrast to 81.3% and 76.3% for upgraded camps and regular camps, respectively. Additionally, pilgrims in the tower class exhibited less involvement in poor hygiene measures, such as consuming expired food, sharing plates and utensils, and sharing water, all of which showed a statistically significant association (p<0.0001).

## Discussion

This study identified a diarrhea prevalence of 32.4% among domestic pilgrims during the 2022 Hajj season, substantially higher than previously reported rates. A 2015 systematic review documented a mean prevalence of 2% (range: 1-23%) [3], while more recent studies from 2016 to 2020 reported rates between 10% and 14% [5,6,8,16]. This three-fold increase compared to historical data and two-fold increase compared to recent pre-pandemic studies warrant the critical examination of potential contributing factors and methodological differences that led to GI symptoms; all reported that approximately 14% of pilgrims had experienced diarrhea [6,8,16].

Several factors may explain this elevated prevalence. First, methodological differences exist between studies: our survey was conducted during the final days of Hajj when recall of recent illness is likely more accurate, potentially capturing cases missed in earlier-departure surveys. However, this timing could also introduce recall bias if pilgrims over-reported mild symptoms. Second, the study design differences, our exclusive focus on domestic pilgrims versus mixed international populations in previous studies, may reflect genuine epidemiological differences, as domestic pilgrims often have different accommodation standards and food sources compared to international pilgrims. Third, our data collection occurred post-pandemic (2022), raising questions about whether temporal trends in food handling practices, crowd density modifications, or other environmental factors have genuinely increased diarrhea risk. However, without longitudinal data or pandemic-specific exposure measurements, we cannot definitively attribute changes to COVID-19-related factors. These alternative explanations underscore the need for standardized surveillance methodology across Hajj seasons to distinguish true prevalence changes from measurement artifacts.

It's believed that hand hygiene plays a critical role as a preventative measure for diarrhea [7,9]. A meta-analysis concluded that handwashing may contribute to a 30% reduction in diarrheal episodes [17]. The findings of this study support this assumption where a statistically significant association was observed between the prevalence of diarrhea and hand hygiene. Pilgrims who cleaned their hands more frequently had lower rates of diarrhea. However, the cross-sectional design of this study precludes establishing causal relationships; we can only report associations, not causation.

Another measure that protects against diarrhea, observed in this study and reported as well in other studies, was the individual use of water, plates, and utensils with no sharing behavior. In 2020, a cross-sectional study with a sample size of 2973 participants with a goal of measuring the effect of personal preventive measures, such as face mask use, hand hygiene, and others, found that the prevalence of diarrhea was significantly associated with poor hygiene-related practices including water, plate, and utensil sharing [18]. These findings may reflect the importance of maintaining good hygiene practices as a preventative measure against diarrhea.

Similar to previous studies, most pilgrims relied on the food provided by the campaign and bottled water as their main sources [5,19,20]. However, pilgrims who relied on other food resources had a higher prevalence of diarrhea. A study conducted in 2008 to correlate diarrhea and sources of food and water mentioned that low standards of food sanitation could be responsible for high rates of diarrhea [8].

A notable finding in this study is the observed variation between campaign classes in terms of the prevalence of diarrhea and its associated symptoms and hygiene practices. To our knowledge, no studies have focused on different campaign classes. Tower class campaigns had the lowest prevalence of diarrhea compared to upgraded camps and regular camps. Associated symptoms were also less prevalent in tower campaigns. To our knowledge, no previous study has taken into consideration the type of campaign when considering similar objectives. These findings support the effectiveness of the strict measures applied by different campaigns. The differences in practices, coupled with relatively less contact and the improved facilities offered in the tower class as well as higher food service standards, may justify the reported discrepancy in symptoms between the campaigns as indicated in previous literature [3-5]. As illustrated in Figure 1, the distribution of diarrhea and its associated symptoms (fever, vomiting, nausea, abdominal pain, and bloating) demonstrates a clear gradient across campaign classes. Notably, tower class pilgrims (orange markers) consistently exhibited the lowest prevalence across all symptom categories, with diarrhea rates approximately 25-30%, compared to regular camp pilgrims (blue markers) who showed rates approaching 40%. This visual representation underscores that the health disparity is not limited to diarrhea alone but extends across the full spectrum of GI manifestations, suggesting systemic differences in exposure risk or underlying health vulnerabilities between accommodation types. Complementing these clinical outcome data, Figure 2 reveals the behavioral underpinnings of these disparities. The figure demonstrates that tower class pilgrims exhibited superior adherence to four critical hygiene practices: hand hygiene (nearly 90% cleaning hands ≥3 times/day vs. 76% in regular camps), avoidance of expired food consumption, minimal water sharing, and reduced sharing of plates and utensils. This concordance between protective behaviors (Figure 2) and health outcomes (Figure 1) provides compelling evidence that the observed differences in diarrhea prevalence are mediated by modifiable behavioral and environmental factors rather than solely reflecting intrinsic population differences. The visual data integration across both figures reinforces that targeted interventions addressing hygiene infrastructure and behavioral practices in lower-tier campaigns could potentially reduce health disparities to levels comparable with tower class facilities.

Beyond Hajj-specific contexts, our findings align with broader patterns observed in mass gathering events globally. Diarrheal illness rates of 20-40% have been documented at other religious mass gatherings, including the Kumbh Mela in India and the Arba'een pilgrimage in Iraq, suggesting that extreme crowding, shared food facilities, and temporary sanitation infrastructure create universal risk conditions. However, direct comparisons are limited by heterogeneity in case definitions, surveillance methods, and population characteristics. Regional studies from other Middle Eastern countries show baseline diarrhea prevalence of 5-15% in community settings, suggesting that the Hajj environment substantially amplifies transmission risk. The observed association between campaign class and diarrhea rates parallels findings from refugee camps and disaster relief settings, where accommodation quality and resource access directly impact enteric disease burden. These parallels underscore that Hajj-related diarrhea is not merely a religious health issue but reflects fundamental principles of environmental health and disease transmission in high-density congregate settings.

Public health implications and recommendations for Hajj health planning

The findings have several actionable implications for Hajj health authorities. First, the substantial disparity in diarrhea rates across campaign classes (tower: lowest; regular: highest) demonstrates that illness is not inevitable but preventable through environmental and infrastructural modifications. Health authorities should prioritize upgrading sanitation facilities, ensuring adequate handwashing stations with soap, and enforcing food safety standards particularly in regular and upgraded campaigns where risk is highest. Second, the strong association between hand hygiene frequency and reduced diarrhea supports scaling up pre-departure health education programs that emphasize specific handwashing techniques (before eating, after toileting) rather than generic hygiene messages. Third, given the elevated prevalence post-pandemic, surveillance systems should be standardized across all campaign types using validated case definitions to enable year-to-year trend monitoring and early outbreak detection. Fourth, the differential risk by accommodation type suggests that resource allocation should be risk-stratified, with enhanced preventive interventions targeted at higher-risk populations. Finally, future Hajj health policies should mandate routine microbiological surveillance to identify predominant pathogens, guide empirical treatment protocols, and inform vaccine development priorities. Implementing these evidence-based strategies could substantially reduce the diarrhea burden and its associated morbidity, healthcare costs, and pilgrim dissatisfaction.

Limitations

Despite the efforts applied to match with the scientific standards in conducting this research, some limitations might be observed. The data collection method, being a survey, introduces the possibility of recall bias as well as self-reporting bias. Another limitation of this study is that the study focused only on domestic pilgrims who might have their own characteristics, which might have affected the prevalence of diarrhea. Further studies on a wide spectrum population might be recommended to come up with more accurate figures about the prevalence of diarrhea and its associated symptoms. Recall bias may have led to over-reporting of symptoms experienced days earlier, while non-response bias could mean that individuals with more severe illness or those who were less concerned about health issues may have been underrepresented. The cross-sectional design prevents us from establishing causal relationships, and the lack of clinical confirmation means that reported "diarrhea" may have included other GI disturbances. These factors collectively suggest that our prevalence estimate should be interpreted with caution and may not reflect the true population burden.

## Conclusions

This study identified a 32.4% prevalence of diarrhea among domestic pilgrims during the 2022 Hajj season. Frequent hand hygiene and appropriate food and water practices were strongly associated with reduced diarrhea rates. Targeted education campaigns focusing on handwashing, safe food handling, and avoiding shared utensils may help reduce illness burden during future Hajj seasons. Specifically, we recommend the following: (1) pre-departure educational materials distributed through Hajj tour operators targeting all domestic pilgrims, emphasizing proper handwashing techniques and food safety; (2) on-site hygiene workshops conducted by health authorities at campaign sites, particularly for regular and upgraded campaign attendees who showed higher diarrhea rates; and (3) provision of accessible handwashing stations and single-use utensils at congregation points and dining facilities. Notably, the 32.4% prevalence observed in this study is substantially higher than the 1-23% range reported in previous literature, suggesting that diarrhea rates among Hajj pilgrims may be increasing rather than previously underestimated.

Further qualitative research is required to provide insight into the discrepancies reported between campaigns. Moreover, we recommend further research projects to investigate the association of diarrhea among different age groups in the context of Hajj, hopefully providing a better understanding of the reported prevalence in comparison to previous studies. Key research questions to explore include the following: (1) "What specific operational differences exist between tower, upgraded, and regular campaign classes that contribute to differential diarrhea rates?", (2) "How do behavioral factors (e.g., handwashing adherence, food sharing practices) vary across campaign types and influence disease transmission?", (3) "What are the age-specific risk factors for diarrhea among Hajj pilgrims, and do these differ by campaign class?", and (4) "How has the prevalence of diarrhea changed over time, and what role do post-pandemic food handling modifications play in observed trends?".
